# How taking pictures of landscapes affects the mental stress of young adults

**DOI:** 10.1002/pchj.773

**Published:** 2024-06-11

**Authors:** Ahmad Hassan, Zhang Deshun

**Affiliations:** ^1^ College of Architecture and Urban Planning Tongji University Shanghai China

**Keywords:** blood pressure, Chinese, landscape, mental health, photography, stress

## Abstract

In today's fast‐paced society, escalating work and academic pressures have led to rising stress levels. While numerous studies have explored adolescent mental health, there has been a lack of focus on “educational stress” among Chinese students. This study sought to understand the psychological and physiological effects of educational stress in Chinese university students. We studied the impact of a 5‐min nature photography session on campus compared with a control activity of photographing urban settings near campus. Data were collected using blood pressure measurements, electroencephalography (EEG), the Semantic Differential Method (SDM), and the State–Trait Anxiety Inventory (STAI) in order to understand psychophysiological reactions. The findings from the SDM and STAI assessments indicated that students felt slightly more at ease and considerably more relaxed, had a heightened sense of naturalness, and experienced reduced anxiety after engaging in nature photography compared with urban photography. Notably, we observed that both systolic and diastolic blood pressure dropped by many values and there were noticeable EEG changes among participants. The results suggest that a brief 5‐min nature photography activity can effectively reduce mental stress in Chinese university students.

## INTRODUCTION

Many people nowadays spend approximately 90% of their time indoors and have substituted outside environments for indoor ones. Most importantly, digital tools such as computers and mobile phones have facilitated communication for those in higher educational settings, and their excessive use has been linked to increased stress and anxiety (Brod, 1984). In addition, many indoor spaces are composed of man‐made objects and prevent people from interacting with nature (Miller, 2005). Furthermore, access to natural communal spaces has decreased as a result of the recent increase in urban density and the expansion of urban workplaces (Fuller & Gaston, 2009; Zhou & Wang, 2011).

In addition, sedentary activities such as surfing the web, watching TV, and playing computer games have cut down on the time people allocate to healthy activities such as spending time in nature. A significant proportion of today's children and adults prefer screen‐based activities during their leisure time, according to a study (Hofferth, 2009). Moreover, factors such as rigorous academic demands on students and parental concerns about safety can inhibit an individual's engagement with the natural world (Lin & Chen, 1995; Valentine & McKendrck, 1997). Many individuals are now disconnected from nature owing to an excessive academic workload, which makes it more likely that they will experience mental health issues such as stress and worry (Miller, 2005; Soga & Gaston, 2016).

Therefore, there is an increasing need for people to connect with nature, and it is becoming increasingly important to prevent mental health problems such as stress. For people to cultivate and cement favorable attitudes toward the environment, regular exposure to natural settings is crucial (Kellert, 2002; Orr & Pyle, 2008); this has been suggested by existing research. For example, according to numerous studies (Bixler et al., 2002; Cheng & Monroe, 2012), early encounters with nature increase a person's affinity for and curiosity about the natural world. Similarly, according to American research by Bixler et al. (2002), those who enjoy natural places typically have more favorable cognitive connections with natural environments. Hinds and Sparks (2008) discovered that adults in the United Kingdom who spent their childhood in rural locations had more positive opinions on nature than those who grew up in metropolitan settings. This shows that an individual's relationship with nature is greatly influenced by their early contact with it (Hinds & Sparks, 2008).

According to recent studies, people are inclined to gravitate toward natural environments because these environments help alleviate stress and counteract attention fatigue (Hassan & Deshun, 2023). Numerous studies have shown that after being exposed to stressful situations, participants who viewed natural landscapes reported fewer negative and more positive emotions than those who observed urban scenes (Herzog et al., 2003; Ulrich et al., 1991). Getting involved with natural areas, and especially with trees, has been linked to enhanced mental well‐being, as this offers solace and calms the mind (Kotera et al., 2020).

There are two main theories, namely the attention restoration theory (ART; Kaplan & Kaplan, 1989) and the stress reduction heory (SRT; Ulrich, 1984; Ulrich et al., 1991), which underpin much of the research in this domain. The ART focuses on cognitive functions, suggesting that activities in natural settings can directly rejuvenate one's attention (Kaplan, 1995; Kaplan & Berman, 2010). Kaplan and Kaplan (1989) state that direct attention requires much mental work, especially when the thing being focused on is unpleasant. This necessitates extra effort to prevent distractions. Overextending this type of attention can lead to fatigue. However, being in a captivating natural setting can replenish or sustain one's attention, invoking a deep sense of relaxation and ease (Kaplan & Kaplan, 1989). Herzog et al. (1997) found that ordinary nature settings helped people recover their attention more fully urban or recreational settings (Herzog et al., 1997).

Numerous studies have confirmed that immersing oneself in nature can aid in alleviating stress and rejuvenating attention capacities (Cimprich, 1993; Hartig et al., 1991; Hartig et al., 2003; Staats et al., 2003; Taylor et al., 2002; Tennessen & Cimprich, 1995; Ulrich, 1984; Wells, 2000). The ART posits that nature possesses elements that naturally captivate us, often referred to as “soft fascination”. Features such as forests, parks, and waterfalls are typically considered softly fascinating, as they effortlessly engage people's attention and evoke positive feelings (Kaplan, 1995; Kaplan & Kaplan, 1989). With their beneficial effects on cognitive processes, working memory, attention regulation, visual focus, vigilance, and cognitive adaptability, natural settings play a pivotal role in renewing our attention (Stevenson et al., 2018). Supporting the ART, research conducted by Ohly et al. (2016) highlighted the remarkably positive effects of nature on individuals, as revealed by a comprehensive review and meta‐analysis of 31 studies (Ohly et al., 2016).

Meanwhile, the SRT outlines the emotional and physiological reactions triggered when one is immersed in a natural environment (Ulrich et al., 1991). Engaging with natural landscapes draws one's attention, shifting negative emotions to positive emotions, thereby helping balance one's physiological systems (Hansmann et al., 2007; Hartig & Staats, 2006). As one's feelings shift toward positivity, they can potentially counteract the cognitive deficits linked to stress (Parsons et al., 1998).

According to Honeyman's 1992 study, photographs of urban regions with and without vegetation as well as photos of rural villages significantly lessen perceived stress. Green urban spaces regularly stood out as a stress‐reducing setting when participants were shown images of these various environments (Honeyman, 1987). Additionally, according to other studies, exposure to natural environments can reduce stress, improve mood, improve physiological well‐being, increase cognitive capacities, and speed up recovery from illness or surgery (Berto, 2005; Knopf, 1987; Leather et al., 1998; Ulrich et al., 1991).

Landscape and urban planning research still lacks thorough evidence‐based studies that explore the psychological and physiological effects of natural versus man‐made settings on photography endeavors, despite some notable findings in this area. Earlier studies mostly used the profile of mood states (POMS) and physiological and psychological tests such as heart rate and heart‐rate variability (Hassan, Tao, et al., 2018). Only a small number, however, have utilized instruments such as the semantic differential method (SDM), the state–trait anxiety inventory (STAI) or electroencephalography (EEG) to explore the real potential of landscape architecture in modern society. The EEG technology stands out as a noninvasive, trustworthy, unfatiguing, and reasonably priced technique. Among other things, it reveals how different sense inputs, such as smell, taste, sight, and sound, affect brainwave activity (Hassan, Qibing, & Tao, 2018). Currently, brain–computer interface research, stress management, anxiety treatment, and other mental health issues all use EEG technology (Nishimura & Mitsukura, 2013).

Electroencephalograms (EEGs) often consist of distinct frequency bands, including delta waves (up to 4 Hz), theta waves (4 to 8 Hz), alpha waves (8 to 13 Hz), beta waves (13 to 30 Hz), and gamma waves (25 to 100 Hz). According to research by Nishimura and Mitsukura (2013), increased cognitive stress or tension correlates with a decline in alpha waves and a corresponding increase in beta waves (Nishimura & Mitsukura, 2013). Similarly, Ajiro et al. (2009) discovered that while alpha waves decreased and theta‐wave activity increased as work pressure or stress increased, some investigations revealed a negative correlation between alpha waves and work‐related stress (Hassan & Zhang, 2023). In a significant study from 1996, Brookings et al. used EEG to look at subtle shifts in workload or stress levels that might elude other assessment techniques. Their study demonstrated how EEG activity patterns can change depending on how an individual interacts with various contexts, highlighting EEG's superiority in accurately capturing human neurophysiological reactions (Brookings et al., 1996).

Many people around the world consider photography during their free time to be a wonderful hobby (Holland, 2021). In Chinese culture, photography has traditionally been highly regarded as a pathway to enhance one's physical, mental, and spiritual well‐being during leisure time (Kent, 2013). Therefore, the purpose of this study is to examine what happens to adults' bodies and minds when they spend time in nature, using photography as a vehicle.

## MATERIALS AND METHODS

### Participants

A total of 28 Chinese university students (males: 18; females: 10; mean age ± *SD*: 21.03 ± 2.23 years old) participated in this experiment. None of them had a record of psychiatric or physical illnesses. The intake of any kind of drug was strictly prohibited. Before the experiment, the methods were explained to the participants, and everyone who participated gave their consent, after having their questions answered. The administrative Ethics Committee of the College of Architecture and Urban Planning, Tongji University China (CAUP23310056) gave permission for this study to be performed.

### Experiment location

**FIGURE 1:**
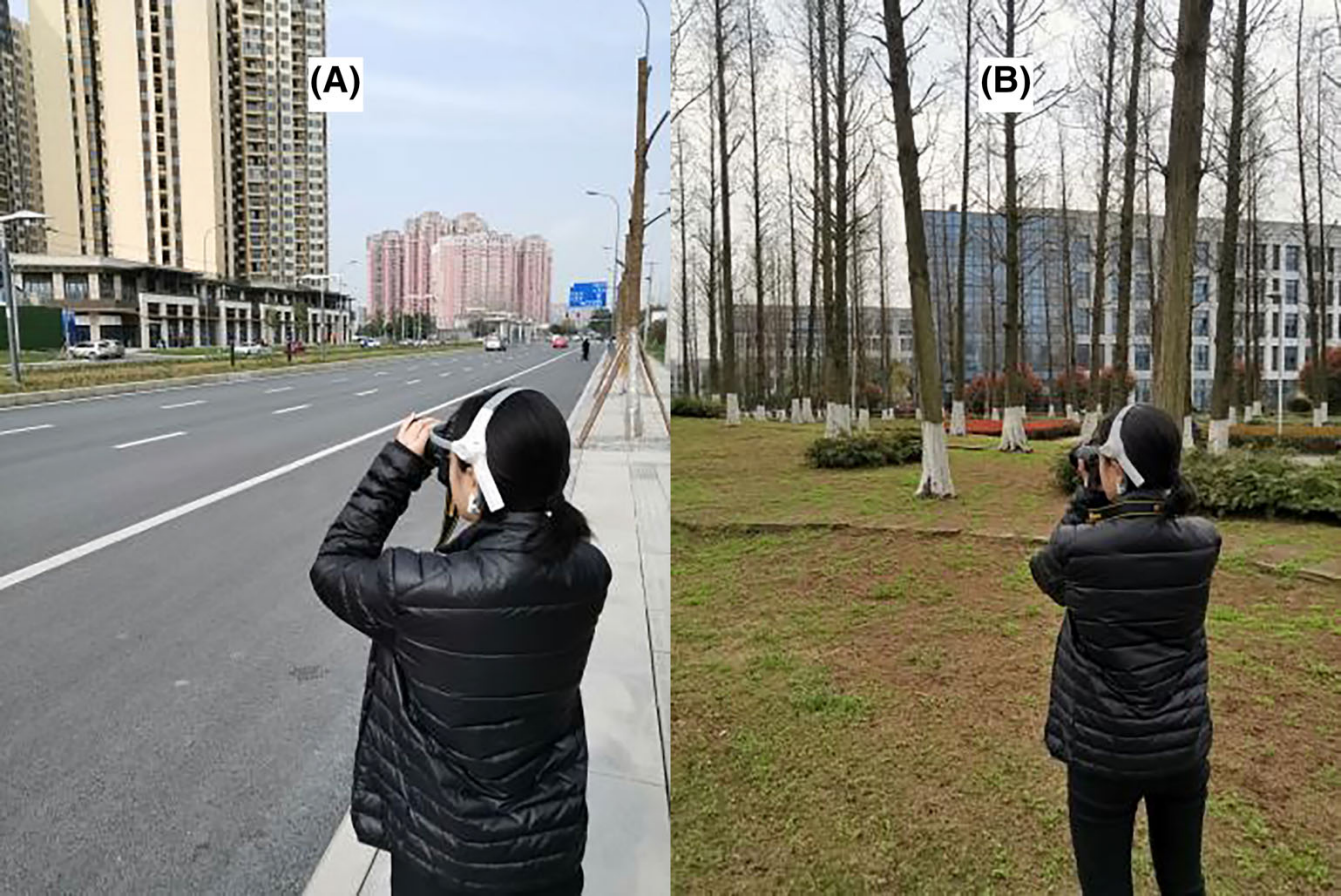
Locations for photography tasks. (A) A participant performing the photography task in an urban area; (B) a participant performing the photography task in nature.

The urban area around Tongji University Siping Road Campus in Shanghai was selected for the control activity (Figure 1). A nature‐based landscape site with many trees and plants, few buildings, and beautiful scenery was chosen as the main experimental site inside the Tongji University Siping Road Campus. The control activity was performed outside the campus, where there was more city traffic and buildings and few trees and plants. For research objectives and participant availability, the experiment was performed on winter days. All of these days were cloudy, and the average temperature and humidity were 22°C and 80%, respectively.

### Protocol

A within‐subject design was used to examine how the events at the two sites affected the body in different ways. Randomly, the 28 people who took part were split into two groups. Group A (14 participants) performed the nature photography task for 5 min in a standing position, while Group B (14 participants) performed the urban photography task (control task) for 5 min in a standing position.

### Measurements

Each participant was directed to the experimental location after receiving an explanation of the experiment in a large living room. The physiological recording equipment included EEG headsets and blood pressure devices. These were fastened to the participant's head and arm, respectively. Participants conducted a nature photography task or an urban photography task (the control) for 5 min while standing after a 5‐min break while sitting down. An Omron sphygmomanometer (HEM‐7011; Omron, Shanghai, China) was used to check the heart rates of the individuals who took part in the study before and after each action. An EEG device called MindWave (NeuroSky Beijing Oriental Creation Technology Co., Ltd, Beijing, China) was used to record EEG data. According to Robbins and Stonehill (2014), this EEG headgear can capture brainwaves from the frontal lobe, which is located above the eyes, in the FP1 region on the human forehead (Robbins & Stonehill, 2014). The four main parts of the headgear are a Bluetooth device, an ear clip, a sensor arm with an EEG electrode on it, and a wristband. There are two dry sensors on the device that are mostly used to find and sort EEG data. Electrical signals from the forehead can be read by the sensor tip. This monitor can also pick up noise from light bulbs, electrical sockets, and other electrical devices, as well as noise from people moving their muscles. Vourvopoulos and Liarokapis (2014) state that the ear clip acts as a base and reference, which allows the chip (ThinkGear) to block electrical noise (Vourvopoulos & Liarokapis, 2014). Along with raw data in the form of a power spectrum (for example, high alpha or beta activity), the device also picks up signs that have to do with concentrating or meditating. Every second, the recorded values are collected, and the raw EEG data are collected at a rate of 512 Hz (Liao et al., 2018). The device also has a small microprocessor that sends and processes electrical signals over Bluetooth and sends them straight to the computer. The raw EEG data showed high alpha and beta activity at 1‐min intervals at each site during the 5‐min task. The overall averages of the two conditions were then compared. The STAI (Kvaal et al., 2005) and SDM (Osgood et al., 1957) questionnaires were used to evaluate psychological status. SDM scores are determined using a 13‐point self‐rating scale based on the following variables: “comfortable/uncomfortable,” “relaxed/awakening,” and “natural/artificial.” Previous research has shown that the SDM is a good and reliable way to measure mood. The 20‐item STAI is a self‐reported questionnaire on a 4‐point Liket scale (1= *almost never*, 4 = *almost always*) to determine how anxious people feel, such as “I feel nervous,” “I feel anxious,” and so on. The STAI scores were made by adding the ratings for the 20 items. Lower scores indicate less anxiety. The individuals who were tested performed both the SDM and the STAI before and after each exercise.

### Statistical analysis

SPSS 16.0 (SPSS Inc., Chicago, IL, USA) was used for statistical analysis. We used repeated‐measures analyses of variance (ANOVAs) and paired *t* tests to examine the physiological differences between the two situations. For physiological data, *p* < .05 was set as the level of statistical significance. Wilcoxon signed‐rank tests with a significance level of *p* < .01 were used to examine the psychological data.

## RESULTS

**FIGURE 2:**
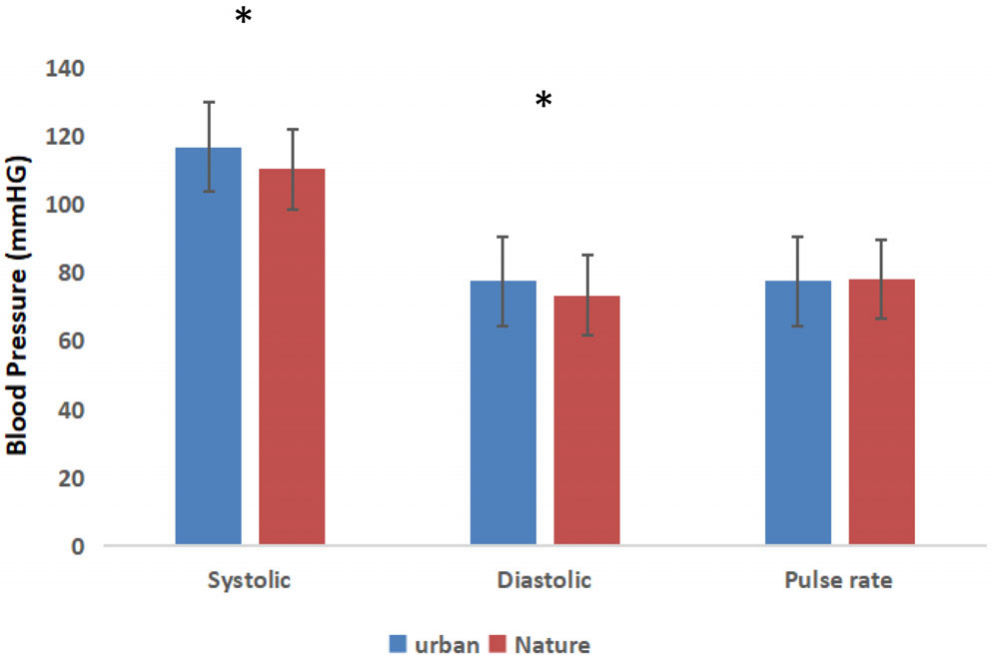
Comparison of blood pressure (mmHg) after performing the photography task in nature and for the control group. *N* = 28; mean ± SD; *p < .05; paired *t* test.

**FIGURE 3:**
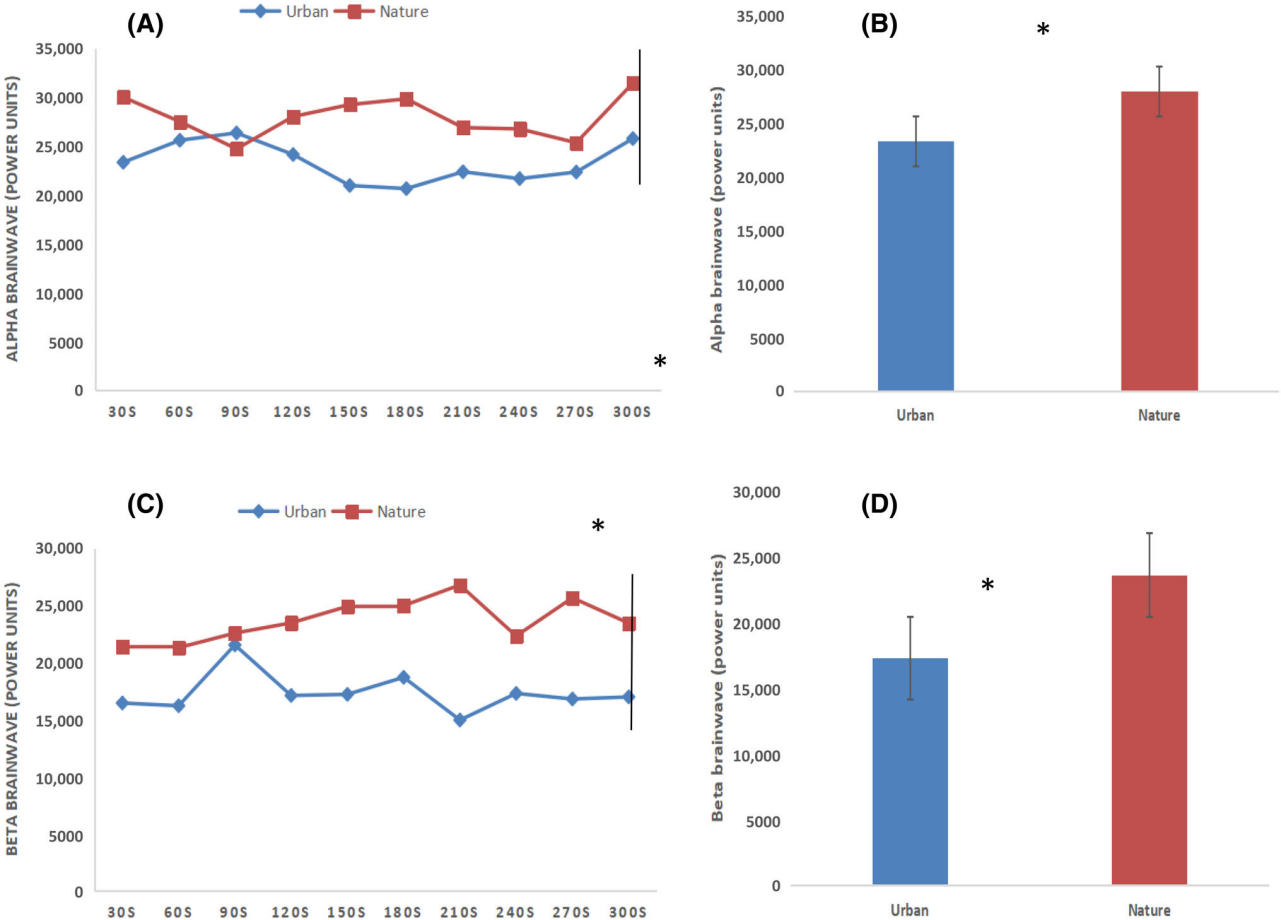
(A) Changes in alpha brainwave power units for the nature and urban groups during a 5‐min photography activity program. A repeated‐measures ANOVA showed a non‐significant time effect (*F* (9,26) = 0.74, *p = *.66) and a significant between‐group effect (*F*(1,26)= 5.61, *p = *.02). (B) Overall mean values for alpha brainwaves. (C) Changes in high beta brainwave power units in nature and urban groups during a 5‐min photography activity program. A repeated‐measures ANOVA showed a non‐significant time effect (*F* (9,26) = 0.5, *p = *.87) and a significant between‐group effect (*F*(1,26)= 5.43, *p = *.02). (D) Overall mean values for beta brainwave power units.

As shown in Figure 2, significant changes in blood pressure were found between the natural and control groups. The mean values (systolic blood pressure, urban: 116.6 ± 9.9 mmHg, natural: 110.2 ± 11.8 mmHg, *p* = .02; diastolic blood pressure, urban: 77.5 ± 4.4 mmHg, natural: 73.3 ± 7.2 mmHg *p* = .01) were significantly lower in participants after 5 min of natural photography than in those who had undertaken urban photography. However, the pulse rate (urban: 77.5 ± 8.6 bpm; natural: 77.9 ± 11.3 bpm; *p = *.88) data indicated no significant changes between the two groups. The participants showed greater changes in alpha (power units) and beta (power units) brainwave patterns after nature photography than after urban photography. In the 30‐s data analysis, most of the alpha brainwave power units increased after natural photography compared with those measured after urban photography (Figure 3A). The alpha brainwave power units over the entire photography period were significantly higher for the nature photography compared with those observed for the control activity (urban: 23276.33 ± 2060.54; nature: 27930.80 ± 2149.75; *p = *.002, Figure 3B). A repeated‐measures ANOVA comparing the mean values of alpha brainwaves (power units) between the nature and control groups with regard to changes in time indicated a significant difference (*F*(1,26) = 5.61, *p = *.02) in relaxation; however, no significant effect of time was found within the groups (*F*(9,26)= 0.74, *p = *.66). Similarly, in the 30‐s analysis, most of the beta brainwave power units increased after nature photography compared with those measured after urban photography (Figure 3C). The beta brainwave power units over the entire photography period were significantly higher in the nature group than in the control group (urban: 17309.41 ± 1753.85; nature: 23668.16 ± 1854.06; *P* < .01, Figure 3D).

**FIGURE 4:**
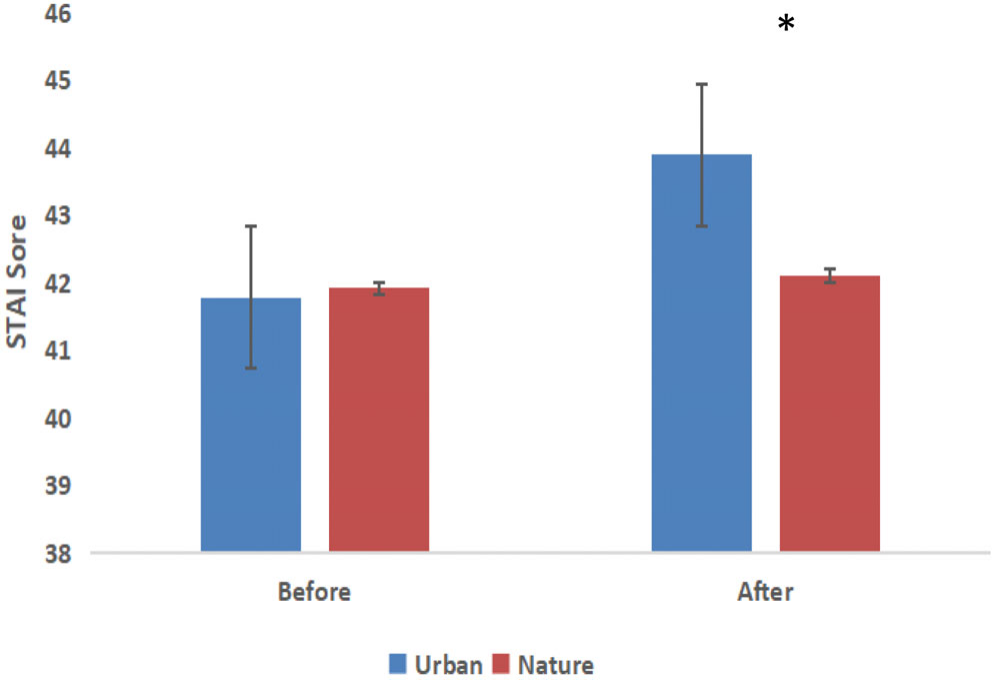
Comparison of STAI scores before and after the photography task in nature and for the control group. *N* = 28; mean ± SE; *p < .05; Wilcoxon signed‐rank test.

**FIGURE 5:**
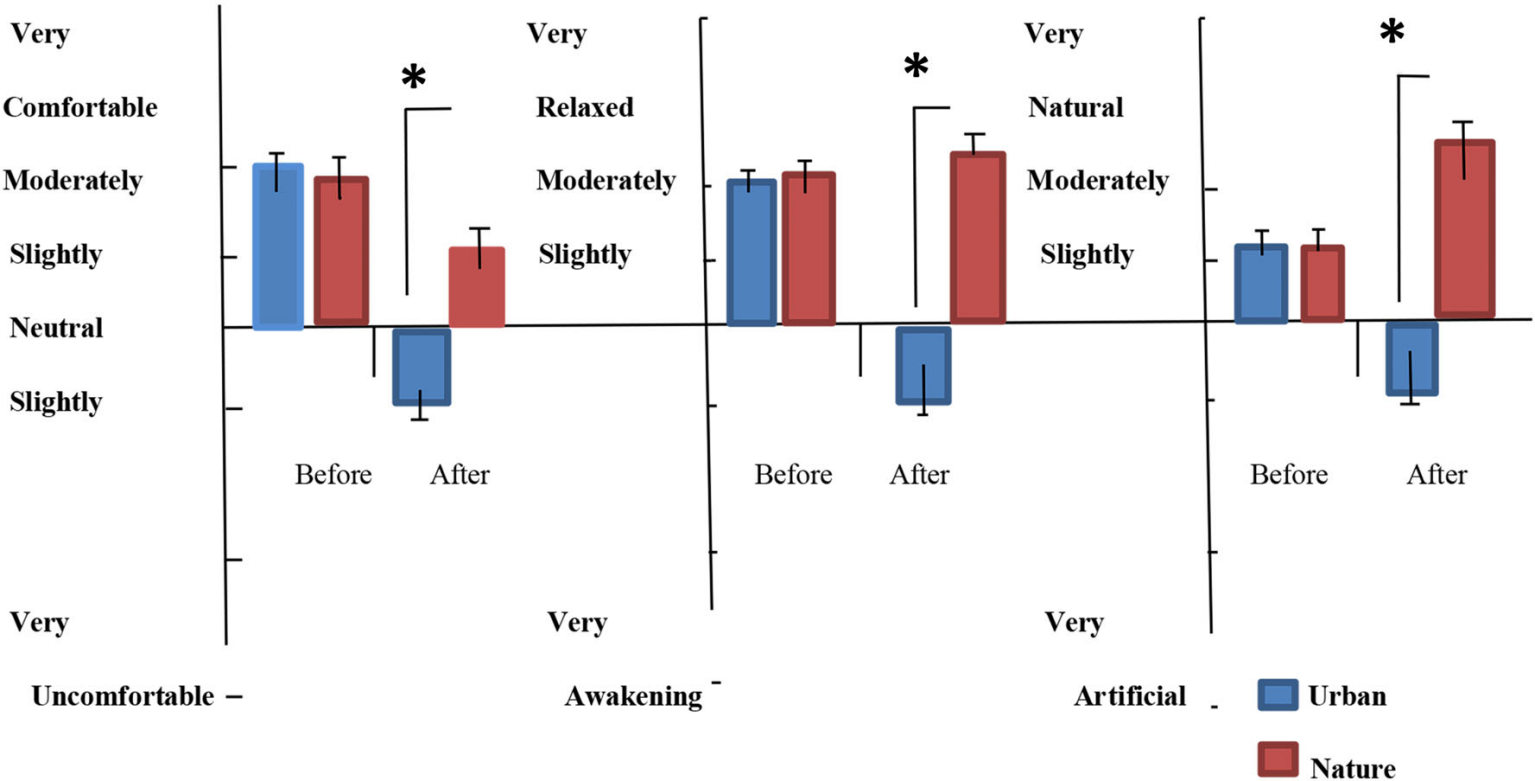
Participant scores for comfortable, relaxed, and natural feelings before and after the tasks. *N* = 28; mean ± SD; *p < .05; Wilcoxon signed‐rank test.

A repeated‐measures ANOVA comparing the mean values of beta brainwaves (power units) between the nature and control groups with regard to changes in time indicated a significant difference (*F* (1,26) = 5.43, *p = *.02) in relaxation; however, no significant effect of time was found within the groups (*F* (9,26) = 0.5, *p = *.87). The STAI and SDM data showed differences between the tasks in the two environments. The overall mean STAI scores significantly decreased (*P* = .02) in the participants after nature photography (42.10 ± 2.84) compared with those measured after urban photography (43.89 ± 2.87) (Figure 4). Furthermore, the participants felt “slightly comfortable,” “moderately relaxed,” and “moderately natural” after performing the photography task in nature compared with after performing the photography task in the urban environment (*p* < .01, Figure 5).

## DISCUSSION

People who live in cities today often feel stressed. The International Labour Organization has identified a number of problems that office workers face, such as more stress, problems with ergonomics, and not having enough time to perform their job (Elsadek & Liu, 2021). Although a number of studies have examined how humans and nature connect (Douglas et al., 2017; Hartig et al., 2014), not much is known about the good links between how taking pictures of landscapes affect the academic stress of young adults. In this study, we examined the stress‐reducing effect of 5 min of photography in natural and urban (control) environments by measuring physiological and psychological variables. Our physiological results indicated that participants in biophilic environments, experienced through undertaking photography in nature, had better stress healing than did the controls over time. Previous research likewise found that people's bodies recovered faster and more completely when they watched videos of natural environments instead of urban ones. For example, skin conductance level (SCL) and pulse transit time (which is linked to SBP) (Parsons et al., 1998).

Additionally, biophilic environments, especially outdoor photography in natural tasks, improved the participants' blood pressure. This is similar to what other research has found: perceiving biophilic environments inside can improve people's blood pressure, especially their diastolic blood pressure (Yin, 2019). Similarly, a systematic review study found that exposure to green spaces outdoors, rather than biophilic environments indoors, was linked to lower systolic and diastolic blood pressure (Twohig‐Bennett & Jones, 2018). A recent randomized controlled experiment that looked at the calming effects of views of a school landscape found that students who had window views of green landscapes recovered much faster from stressful events. The researchers in that study measured the students’ short‐term Heart rate variability (low‐frequency/high‐frequency) and SCL (Li & Sullivan, 2016). Additionally, the results of the current study agree with those of a previous study we performed indoors on how different visual scenery stimuli affected Chinese students' stress levels. In this previous study, both systolic and diastolic blood pressure in the students decreased after 110 s of looking at photos of various types of scenery instead of at photos of traffic in cities (Jiang et al., 2020). The SRT posits that looking at natural settings can lower stress and negative emotions because our bodies have evolved to naturally prefer those places. This fits with what we found about how the body reacts during the restoration process (Ulrich et al., 1991). Additionally, the four physiological measures—heart rate variability, heart rate, SCL, and blood pressure—show activity in various body systems that are all connected to the autonomic nervous system. The stronger SRT that biophilic environments could help lower physiological stress levels was shown by the consistent trends across these physiological reactions. Therefore, we can say that their lower blood pressure indicated that adults who interacted with nature through photography tasks were healthier than adults who performed the control task. In this experiment, we investigated participants' stress by examining the impact of natural and urban photography environment exposure on human brain activity. Hence, in this study, we investigated the following research questions. Do nature and the urban environments differ significantly in the statistics of photography? Do human brains act differently in these different environments? What are the significant differences in the alpha and beta brainwaves when responding to the two different types of environmental stimuli?

The EEG scan is a new way for scientists to track how stressed people are. It shows how changes in normal brainwave patterns can be caused by things outside the person's head (Jing & Takigawa, 2000). For instance, participants’ high levels of activity in the alpha and beta frequency ranges of brainwaves showed that the nature photography task made them feel less stressed, while the urban photography task made them feel more stressed. After 5 min of taking pictures of nature, the participants' higher alpha waves showed that the task had made them feel less stressed. Therefore, the fact that alpha waves increased in the nature photography task group showed that participants were more relaxed during this work (Başar, 2012; Neuper & Pfurtscheller, 2001), and the lower alpha waves in the control group showed that participants in that group were stressed during the task. Researchers have found that alpha waves can help people feel less stressed and remember things better (AlShorman et al., 2020). The findings of this study are in line with those found in a prior investigation conducted by Hassan et al., which found that strolling in an environment consisting of bamboo forests had a significantly increased alpha brainwave activity but that this decreased in a city environment (Hassan, Tao, et al., 2018). Furthermore, various studies have demonstrated that being in nature increases the strength of alpha waves, which are linked to relaxation and healing in the body (Alarcao & Fonseca, 2017; Kim et al., 2013; Ursuţiu et al., 2018). This result is similar to the findings of Kim et al. (2016). These authors reported that individuals’ alpha waves greatly increased after smelling an extract of orchid flowers (Kim et al., 2016). The individuals who took part in the 5‐min photography task had increased alpha brainwaves (power units) as soon as they began taking pictures outside. However, when the participants started the photography task in the city area, their high alpha brainwaves decreased. The results agree with those of a previous study that looked at how photos of different types of scenery (visual stimuli) viewed for 110 s affected the mental and physical health of Chinese students (Jiang et al., 2020). The results show that the ratio of the alpha band increased as calmness increased. Although our results are still preliminary, they show that the 5‐min nature photography task may have made the participants more relaxed and helped them recover to a greater extent compared with those in the control group. Because of this, one suggestion from this finding is to use photography, for example, places that need to be calm, such as school gardens, parks, and home lawns.

For the same reason, the participants' beta‐wave activity increased after 5 min of the nature photography task. This means that those in the nature photography task group were able to relax and focus better, and their mental sharpness was better compared with the control group. Studies in the past have shown that the appearance and changing of beta waves show how focused and attentive someone is (Coben et al., 2010). For example, when people looked at pictures of natural scenery, their beta waves became stronger, and their energy and focus increased (Jiang et al., 2020). The results of our study are consistent with previous studies of EEG data showing that physiological reactions are different in different seasonal landscapes and affect beta brainwaves (Wang & Xu, 2021). Furthermore, a total of five studies, including the effects of a green wall compared with a non‐green wall, indoor plants in the working environment, fragrant primula flowers, gardening activity, and the greenness of an interior space, were found to raise alpha and beta waves, which reveals less mental stress (Gu et al., 2022). An increase in beta power has been observed during rest (Vijayalakshmi et al., 2010). Most of the time, strong feelings such as excitement, focus, and attention are linked to beta waves (Neuper & Pfurtscheller, 2001). Individuals who have increased beta waves have been observed to have better focus and mental sharpness (Neuper & Pfurtscheller, 2001). Low beta‐wave activity can also lead to large changes in mood (insomnia) (Hauri, 1981). When one is alert or focused on a mental task, beta waves are the strongest waves. During sleepiness, on the other hand, beta waves decrease (Hassan, Qibing, & Tao, 2018). Beta waves occur naturally during many activities, such as having deep talks, playing sports, listening to speeches, and going to class to hear lectures (Wolfe, 2010). The results of our study showed that having increased beta waves was linked to better attention. As a result, it might be suggested that a 5‐min photography task be used in the classroom or workplace to help students or employees focus better and get more done. The findings of both the STAI and the SDM suggest that photography in natural settings has beneficial effects on mental stress, in contrast that in urban environments, which has been shown to have detrimental effects on mental stress. Previous psychological studies, for example on the effects of a green wall versus a non‐green wall on occupants, a small plant inside on the desk (Yeom et al., 2021), what plants can do for young adults who are stressed (Hassan, Qibing, Tao, et al., 2018), and how blooming and leafy plants in hospital rooms help people who have just had abdominal surgery (Park & Mattson, 2008), have shown that exposure to small numbers of green plants is linked to large decreases in worry and mental stress. One study was performed on a specific group of people: those who had an appendectomy. The results showed that having plants in the room helped the patients heal after surgery. The improvement effect of a large indoor green wall was much lower than that of a small green wall, according to Yeom et al. (2021). A small green wall led to a small drop in state anxiety. In Ke‐Tsung Han's study, adding six pots of leafy plants to a classroom immediately made people feel less anxious. However, the effect faded over time and became statistically unimportant after a while (Gu et al., 2022). The effects of the natural environment have been extensively researched and shown to promote stress reduction and attention restoration compared with urban environments (Sugiyama & Thompson, 2006).

The Psycho‐physiological stress reduction theory and ART provide a theoretical basis for the calming effect of interacting with natural and urban greenery. They suggest that contact with nature can help people who are under a lot of stress by making them feel better and that the unconscious attention that people pay to interesting and rich stimuli in nature can help them do better on mentally demanding tasks. Research has shown that exposure to natural environments, such as through photography, can help people to become less stressed and pay attention again, thereby explaining the different results observed in natural and urban environments (Ohly et al., 2016). The fact that participants' systolic and diastolic blood pressure dropped significantly and their EEG patterns changed when they spent 5 min taking photos in natural versus urban settings supports the idea that nature can help reduce stress and improve attention. There may be a key role for aesthetic factors, such as greenery and natural surroundings, in both natural and seminatural urban settings in making the healing effects stronger and lowering mental stress. Additionally, Kaplan's ART says that looking at nature can help the brain heal from mental fatigue and return to paying attention. This could explain the noticeable EEG changes that were observed in people who were in nature. The participants who took photos for 5 min in nature had significantly lower systolic and diastolic blood pressure levels and noticeable EEG changes compared with those who took photos in cities. This is likely because nature has healing effects on the mind and body. Exposure to natural settings, such as through photography, has been shown to help reduce stress and improve attention. This explains why the effects are different in nature compared with cities. The benefits of 5‐min photography in nature include that it is an easy, feasible, and useful way for people in cities to improve their health and quality of life. Nature's benefits are very important for mental health, so city planners and landscape artists should work to keep green spaces in cities to improve people's lives.

However, this study has some limitations. For example, only a few young men and women were asked to participate. To fully understand how brainwaves work, more research with large sample sizes during different growth seasons, such as summer and spring, is needed. Additionally, more in‐depth studies need to be performed in a variety of natural settings, such as forests, parks, and gardens.

## CONCLUSIONS

Our study suggests that performing a photography task in nature for 5 min could lower psychophysiological stress to a greater extent than performing a photography task in an urban area.

## FUNDING INFORMATION

The National Natural Science Foundation of China (32071824) supported this research.

## CONFLICT OF INTEREST STATEMENT

Each author has confirmed that they have no conflicts of interest.

## ETHICS STATEMENT

Subject gave written informed consent before any tests were performed. The research was conducted at Tongji University in China with the consent of the school's Ethics Committee for the College of Architecture and Urban Planning.

## CONSENT FOR PUBLICATION

Particpants provided written informed consent for publishing, and permission to publish photos was also acquired.

## Data Availability

The data used to support the results of this study are proprietary and hence unavailable.
